# Stretchable, Self-Healing, and Processable Bioelastomers Enabled by Highly Branched Cross-Linking Networks for Flexible Electronics

**DOI:** 10.34133/research.1391

**Published:** 2026-08-05

**Authors:** Yuan Liu, Xin Xu, Jinsong Sun, Aojing Xue, Jianing Liu, Yuhan Lou, Jiajun Liu, Shi Liu, Dan Sui, Zhiyi Hou, Gegu Chen, Luyao Wang, Yongzhuang Liu, Dawei Zhao, Haipeng Yu, Qinqin Xia

**Affiliations:** ^1^ State Key Laboratory of Woody Oil Resources Utilization, Northeast Forestry University, Harbin 150040, P.R. China.; ^2^ Analysis and Testing Center, Northeast Forestry University, Harbin 150040, P.R. China.; ^3^ MOE Engineering Research Center of Forestry Biomass Materials and Bioenergy, Beijing Forestry University, Beijing 100083, P.R. China.; ^4^ Key Laboratory on Resources Chemicals and Materials of Ministry of Education, Shenyang University of Chemical Technology, Shenyang 110142, P.R. China.

## Abstract

Stretchable elastomers require a stretchability exceeding 200% and a Young’s modulus ranging from 0.1 to 10 MPa to balance strength and extensibility for dynamic applications. Conventional petrochemical polymers meeting these criteria rely on covalent networks, which lack mechanical tunability and face challenges in terms of repairability and recyclability. Here, we introduce a highly dynamic covalent branched network to fabricate a polyimine elastomer (PIE) featuring extensive mechanical tunability, controllable self-healing properties, reprocessability, and recyclability. This PIE is synthesized at room temperature from a lignocellulose-derived dialdehyde prepolymer (dialdehyde-terminated prepolymer) and cross-linked with a triamine via imine bonds. The resulting PIE, characterized by dynamic covalent networks, presents a skin-friendly Young’s modulus of less than 3 MPa and an impressive stretchability of strain exceeding 1,110%. Additionally, PIE exhibits direct thermal reprocessability at 100 °C within 15 min, enabling transformation into various forms, along with self-healing capability. Flexible electronics utilizing the PIE substrate demonstrate appealing stretchability, high sensitivity, and recyclability. This biomass-derived highly branched cross-linking network likely provides a promising approach for designing high-performance bio-based synthetic elastomers, contributing to the advancement of sustainable flexible electronic devices.

## Introduction

**Fig. 1.**
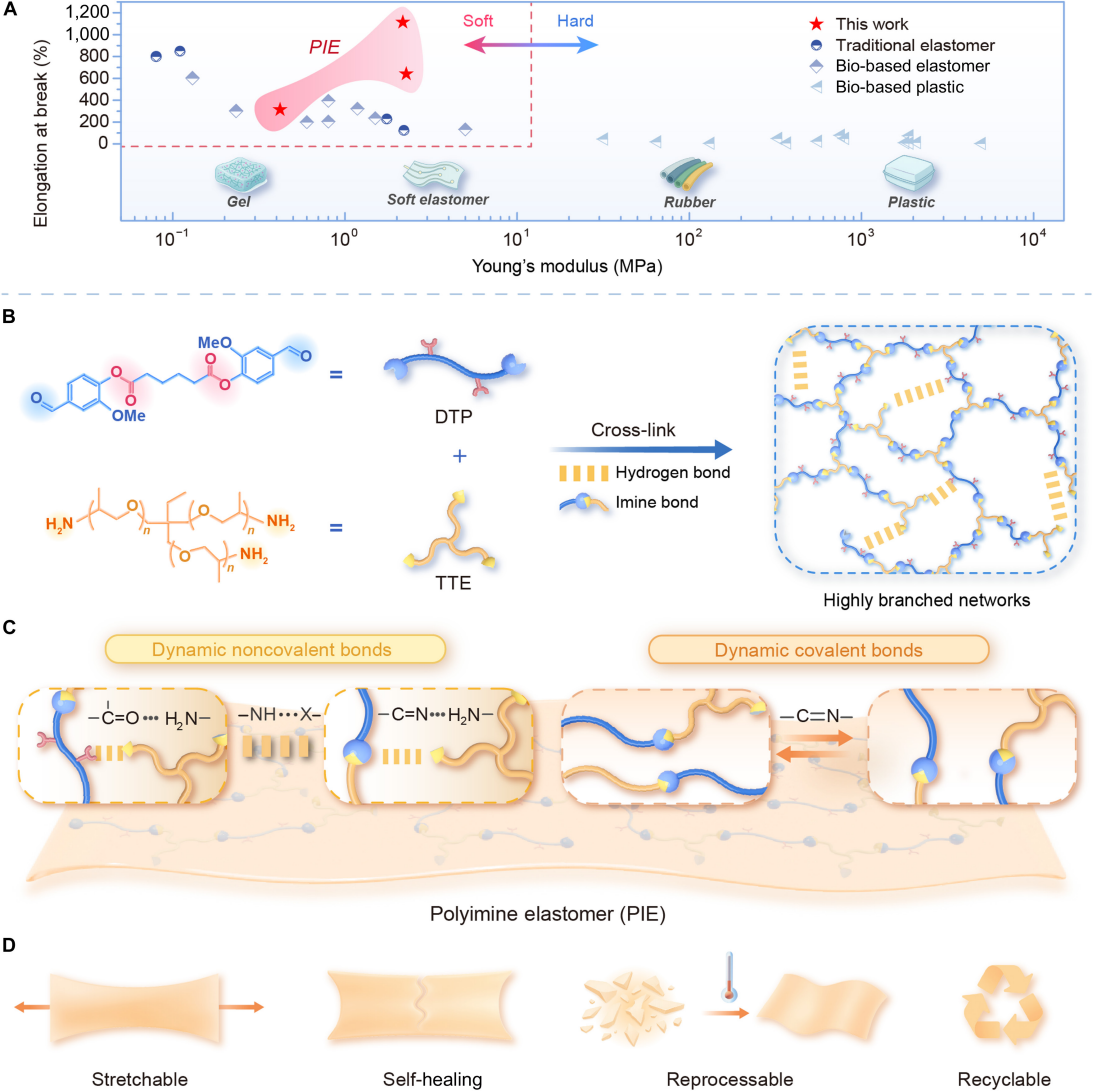
Design concept of highly branched polyimine elastomers (PIEs). (A) Mechanical properties of some representative polymers, including traditional elastomers, bio-based elastomers, and bio-based plastics. (B) Synthetic route to the dialdehyde-terminated prepolymer (DTP)-derived highly branched PIE network. (C) Supramolecular and dynamic covalent interactions within the PIE network. (D) Integrated properties of PIE, including stretchability, self-healing, thermal reprocessability, and chemical recyclability.

Stretchable elastomers, characterized by intrinsic compliance and elasticity (Young’s modulus 0.1 to 10 MPa; elongation > 200%) [1,2], are essential for developing flexible and wearable electronics [3–5] that can adapt to dynamic interfaces and large-strain deformation [6–8] (Fig. 1A) [9–19]. Conventional petroleum-based elastomers rely on permanent cross-links to meet basic mechanical needs, at the expense of their repairability and recyclability. Dynamic covalent bonds (e.g., imine bonds [20,21], transesterification [22,23], and disulfide bonds [13,24,25]) can be incorporated into stretchable elastomers to impart repairability and recyclability through reversible bond exchange. However, the use of petroleum-derived precursors and intricate syntheses presents a key challenge for improving elastomer sustainability and practicality.

Advances in biorefinery enable the conversion of lignocellulosic biomass [26–29]—an abundant and renewable resource—into key platform chemicals [30], including vanillin (VA), lactic acid, and various dicarboxylic acids, such as succinic acid and adipic acid (AA). These compounds offer both inherent sustainability and versatile reactivity, serving as crucial building blocks for sustainable stretchable elastomers. Several of them are already produced at an industrial scale (e.g., VA at ~3,000 t/year [31] and bio-based AA at ~3.3 million t/year [32]). This ready availability highlights the feasibility of developing scalable and sustainable elastomer systems based on biomass-derived feedstocks. A notable example is polyimine, synthesized via a straightforward Schiff base reaction between VA and amines. While they offer facile production and can be effectively processed and recycled under mild conditions (<100 °C) via bond exchange and hydrolysis [21], these materials typically exhibit poor mechanical performance, particularly limited stretchability with elongation at break often below 12% [33]. Alternatively, converting VA into prepolymers through prior chemical modification (e.g., alkyl halide grafting [34,35] or methacrylic anhydride functionalization [36]) increases the cross-linking density, thereby enhancing mechanical strength and achieving elongations at break reaching up to ~60% [37]. However, these systems generally suffer from complex synthetic routes and relatively harsh reaction conditions (e.g., multistep functionalization, the use of toxic chemicals, or elevated reaction temperatures). More importantly, polymerization is often limited and poorly controlled, which restricts polymer-chain mobility and ultimately leads to limited stretchability of polyimine materials. Therefore, developing bio-based polyimine elastomers (PIEs) with integrated controllable structures, high stretchability, and dynamic reconfigurability via a simple yet efficient polymerization method remains a substantial challenge.

Here, we report a lignocellulose-derived highly branched PIE. Its structure, which incorporates imine bonds and a tunable branching architecture, is achieved simultaneously by simply adjusting the monomer feeding ratio. A dialdehyde prepolymer (dialdehyde-terminated prepolymer [DTP]) is first synthesized from VA and AA at room temperature, followed by polycondensation with a triamine to construct a highly branched polyimine network (Fig. 1B). Within this network (Fig. 1C), cooperative dynamic interactions arise from hydrogen bonding between free amines and ester/imine groups, together with reversible imine exchange. This highly branched architecture endows the PIE with exceptional stretchability and mechanically tunable properties (Young’s modulus of less than 3 MPa), with elongation at break ranging from 314.1% to 1,113.3%. In addition, the dynamic covalent bonds enable self-healing, thermal reprocessability, and chemical recyclability (Fig. 1D). Owing to its intrinsic softness and interfacial adaptability, the PIE enables seamless integration with conductive inks for high-performance sensing. The simplicity, scalability, and mild reaction conditions of this strategy provide a sustainable platform for next-generation flexible and stretchable elastomers.

## Results and Discussion

### Synthesis and characterization of the prepolymer and PIE

**Fig. 2.**
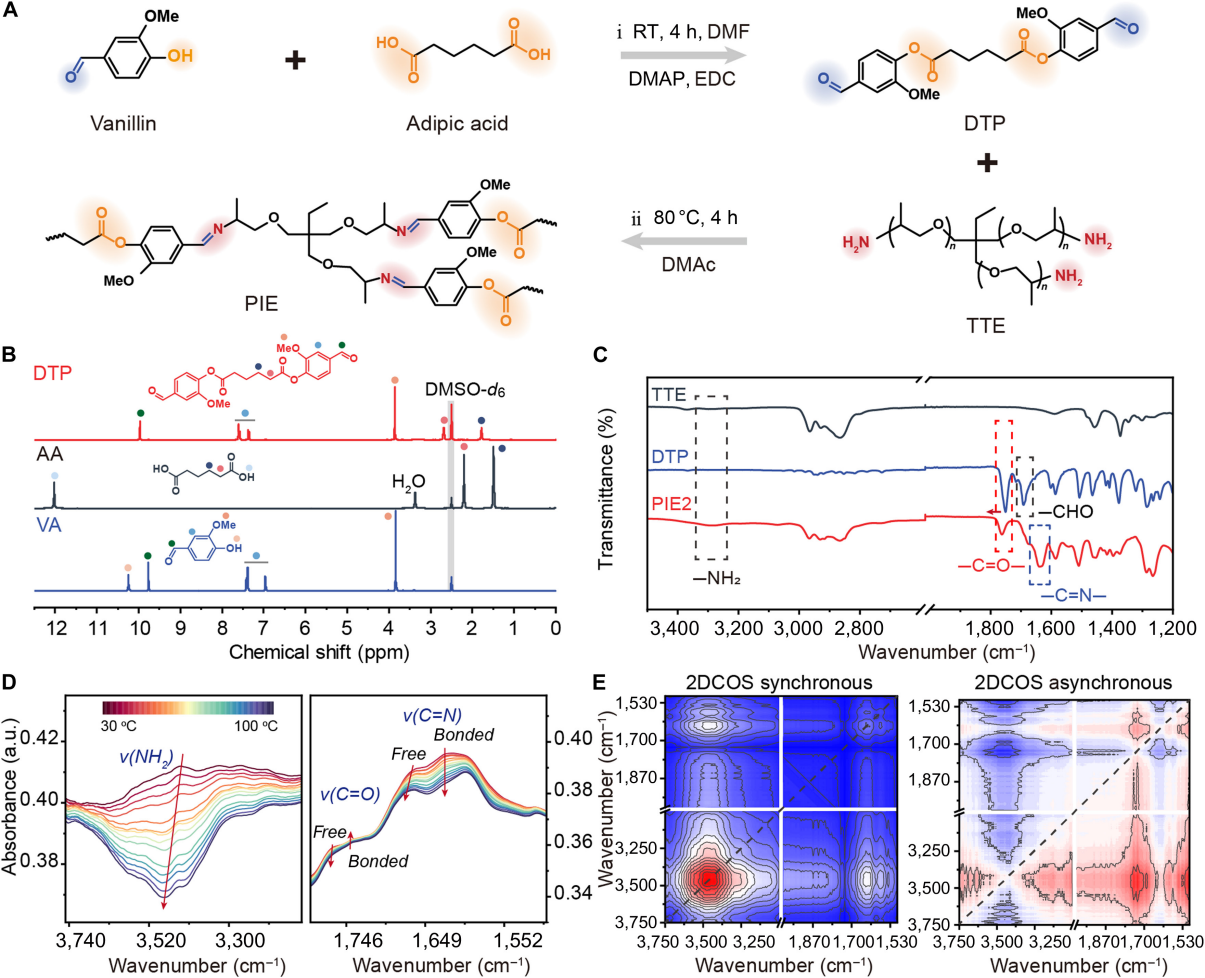
Synthesis and characterization of dialdehyde-terminated prepolymer (DTP) and polyimine elastomer (PIE). (A) Synthetic routes of (i) DTP and (ii) PIE. (B) Proton nuclear magnetic resonance (^1^H NMR) results of DTP. (C) Fourier transform infrared (FT-IR) spectra of trimethylolpropane tris[poly(propylene glycol), amine terminated] ether (TTE), DTP, and PIE2. (D) Variable-temperature FT-IR spectra of PIE2. (E) Synchronous and asynchronous 2-dimensional correlation infrared (2D-COS-IR) spectra of PIE2.

Lignocellulose-derived monomers inherently offer sustainability and diverse active functional groups, enabling the synthesis of high-performance bio-based elastomers. The direct polymerization of bioelastomers often presents undesirable cross-linking [38], uncontrolled degrees of polymerization, and limited chain slippage [39] due to the small dimensions of the constituent monomer units. To provide controllable cross-linking sites and segmental mobility, we designed a DTP from 2 readily available, lignocellulose-derived platform chemicals: VA and AA. The reaction of VA and AA at a 2:1 molar ratio at room temperature yielded DTP, which was subsequently precipitated by the addition of water. The resulting DTP possesses a well-defined symmetric structure, cloaked in internal ester linkages and crowned with reactive aldehyde end groups. This key motif enables its reaction with triamine (trimethylolpropane tris[poly(propylene glycol), amine terminated] ether [TTE]) at 80 °C for 4 h to produce a highly branched PIE, which undergoes cross-linking through dynamic imine bonds (Fig. 2A).

The chemical structure of DTP was confirmed by proton nuclear magnetic resonance (^1^H NMR) analyses (Fig. 2B). The disappearance of the phenolic OH (ArOH) signal of VA at *δ* 10.24 ppm and the carboxyl (–COOH) signal of AA at *δ* 12.02 ppm confirm the occurrence of esterification. In addition, the methylene proton signals of AA (*δ* 2.19 and 1.48 ppm) display pronounced chemical-shift variations, whereas the aromatic (*δ* 7 to 8 ppm) and methoxy (*δ* 3.85 ppm) resonances of VA remain essentially unchanged. Notably, the integrated peak areas closely match the theoretical proton ratio, thereby supporting the proposed molecular structure and indicating the high chemical purity of the resulting DTP (Fig. S1). Fourier transform infrared (FT-IR) spectroscopy (Fig. 2C) showed a distinct absorption peak at 1,750 cm^−1^ corresponding to the C=O stretching vibration, indicative of ester-bond formation. Following the Schiff base reaction between TTE and DTP, the aldehyde (–CHO) peak of DTP at 1,690 cm^−1^ disappears, while a new peak appears at 1,640 cm^−1^, corresponding to the formation of the imine bonds (–C=N–) in PIE2. Furthermore, the characteristic peaks observed in the high-resolution x-ray photoelectron spectroscopy at 285.3 eV for C 1s and 400.2 eV for N 1s further confirm the formation of imine bonds of PIE2 (Fig. S2).

PIE2 contains free amino groups (broad FT-IR band at 3,300 cm^−1^) that act as hydrogen-bond donors to the ester, imine, and ether groups (acceptors). The ester (–C=O) groups from the DTP segment also act as hydrogen-bond acceptors, contributing to the hydrogen-bonding interactions within the PIE network. Variable-temperature FT-IR (Fig. 2D) reveals the progressive dissociation of C=N···H–N interactions upon heating, marked by a blueshift and narrowing of the N–H stretching band (3,200 to 3,500 cm^−1^), weakening of the hydrogen-bonded ν(C=N) band at 1,625 cm^−1^, and the enhancement and blueshifting of the free ν(C=N) band at 1,660 to 1,675 cm^−1^. Meanwhile, the ν(C=O) band at 1,761 cm^−1^ exhibits a slight redshift and develops a shoulder near 1,748 cm^−1^, suggesting the formation of new C=O···H–N hydrogen bonds as the temperature increases. These spectral changes demonstrate a temperature-induced redistribution of hydrogen donors between imine and carbonyl acceptors, with C=O···H–N interactions being energetically stronger, consistent with the results of 2-dimensional correlation infrared spectroscopy [40] (Fig. 2E and Table S1). In addition, the PIEs are optically transparent and exhibit smooth surfaces under scanning electron microscopy, while their x-ray diffraction patterns show no distinct diffraction peaks (Figs. S3 and S4), confirming an amorphous structure that favors subsequent segmental mobility within the network.

### Mechanical properties of PIE

**Fig. 3.**
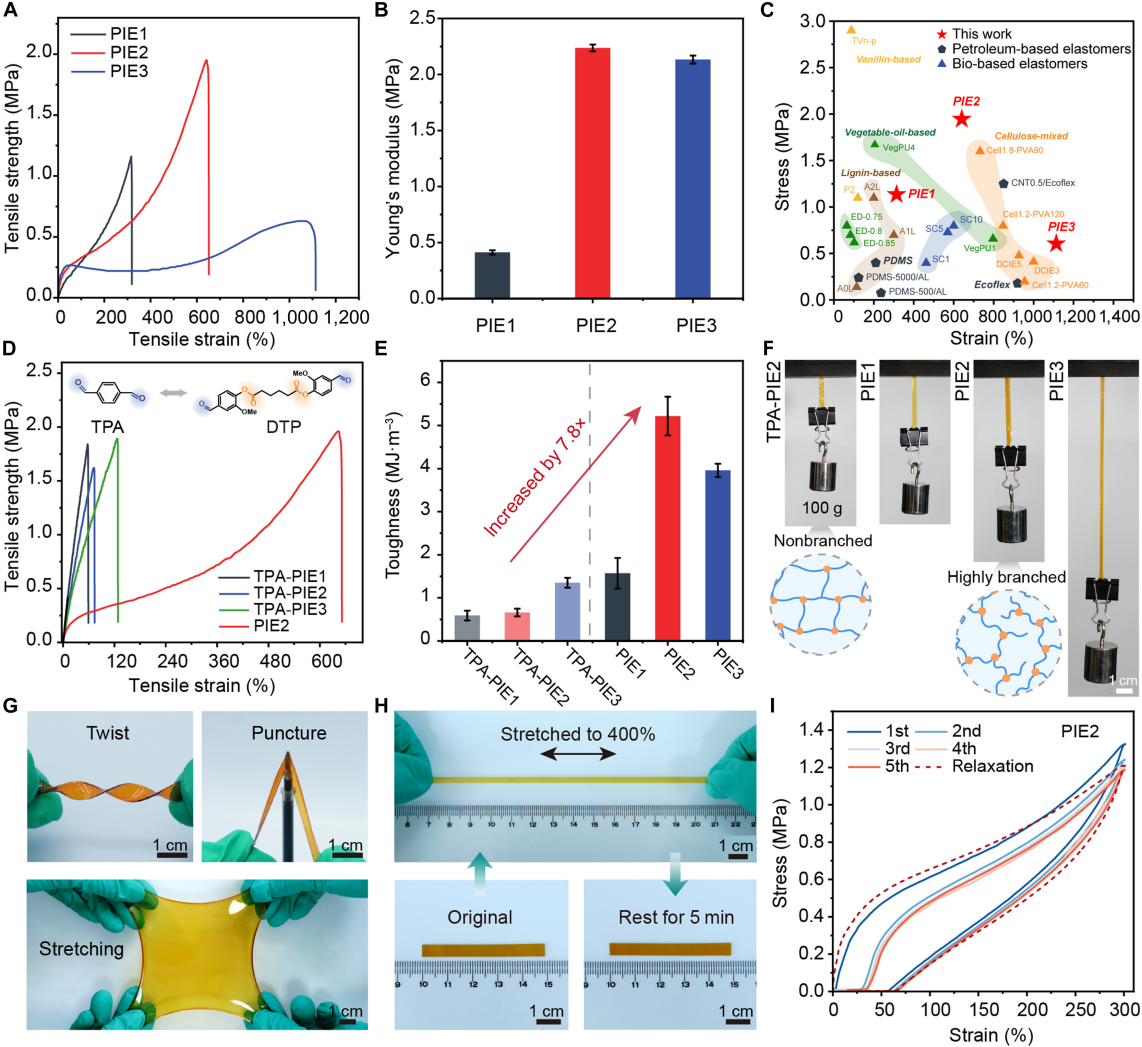
Mechanical properties of polyimine elastomer (PIE). (A) Stress–strain curves of PIE. (B) Young’s modulus of PIE. (C) Comparison of the stress and strain of PIE with those of other reported elastomers. (D) Stress–strain curves of terephthalaldehyde (TPA)-PIE and PIE2. (E) Toughness of TPA-PIE and PIE. (F) Optical images of PIE and TPA-PIE2 supporting a 100-g weight. (G) Optical image of the twisted, flexible PIE2. (H) Optical image of a PIE2 specimen after recovery from 400% stretching. (I) Cyclic tensile curve of PIE2.

Broad mechanical tunability is a critical requirement for elastomers, as it enables adaptation to diverse deformation modes and application scenarios. By integrating multiple hydrogen-bonding interactions with a highly branched architecture, the PIE network achieves exceptional mechanical tunability. Specifically, 3 polyimine networks (PIE1, PIE2, and PIE3) were synthesized by varying the DTP/TTE ratio to 3:2, 6:5, and 1:1, respectively (Fig. S5). The stoichiometric 3:2 ratio balances functional groups, whereas increasing the TTE content introduces more dynamic cross-linking sites and tailors the branching density. As shown in Fig. 3A, the mechanical behavior of PIE is highly tunable: the elongation at break increased from 314.1% for PIE1 to 1,113.3% for PIE3. A moderate increase in TTE promotes network branching and strengthens hydrogen-bonding interactions, thereby enhancing chain mobility and energy dissipation. In contrast, the tensile strength exhibits a nonmonotonic trend. It initially increases from 1.14 MPa for PIE1 to 1.95 MPa for PIE2, followed by a decrease to 0.61 MPa for PIE3 at the highest TTE content. This behavior can be attributed to excessive branching, which enhances chain flexibility and energy dissipation but simultaneously lowers the effective cross-linking density and weakens the hydrogen-bonded network. The corresponding Young’s modulus values, 0.42 MPa for PIE1, 2.27 MPa for PIE2, and 2.17 MPa for PIE3, remain within the range typical of soft elastomers (Young’s modulus: 0.1 to 10 MPa; elongation > 200%) (Fig. 3B). Our PIE outperforms a broad range of elastomers in overall mechanical tunability, including commercial elastomers such as polydimethylsiloxane and Ecoflex [10,41–43], as well as most reported bio-based counterparts [5,12,13,24,44–47] (Fig. 3C and Table S2).

Dynamic imine bonds combined with a highly branched spatial architecture enable effective regulation of the mechanical properties of the elastomeric network, in which the chain length of the DTP motif plays a critical role in promoting a highly branched structure and network flexibility. To elucidate the structural advantage of the DTP motif, a control sample (denoted as TPA-PIE) was prepared by replacing DTP with short-chain terephthalaldehyde (TPA), which reacts with imine-forming amines under identical conditions. Owing to its confined molecular geometry and limited chain length, the TPA-based system undergoes rapid local cross-linking, leading to the formation of a rigid network with severely restricted extensibility. In contrast, PIE2 exhibited an elongation at break 8.7-fold higher than that of TPA-PIE2 (640.6% vs. 73.6%), alongside a 7.8-fold improvement in toughness (4.89 MJ·m^−3^ vs. 0.62 MJ·m^−3^) (Fig. 3D and E). The distinct elongation behaviors of PIE and TPA-PIE were further confirmed by the hanging-weight deformation test (Fig. 3F). The highly branched structure of DTP provides the molecular flexibility and spatial reach that enable effective connection of TTE amines with adjacent DTP units, thus favoring extended branching over premature cross-linking. This mechanism is inherently more conducive to constructing the soft, highly branched elastomeric networks essential for advanced applications.

The PIE materials display a compelling combination of flexibility, puncture resistance, and stretchability (Fig. 3G). Among them, PIE2 exhibited the highest puncture force of 12 N, which is consistent with its superior tensile strength (Fig. S6). After being stretched to 400% strain, PIE rapidly recovered nearly to its original length (Fig. 3H). Cyclic tensile tests (Fig. 3I) revealed pronounced energy dissipation and initial residual strain, behaviors attributed to the rupture of hydrogen-bonded segments and interchain friction. The material gradually regained its original strength through hydrogen-bond reformation. The gradually enlarged hysteresis loops with increasing strain (Fig. S7) further confirm effective energy dissipation and the reversible nature of the internal supramolecular interactions through dynamic hydrogen bonding. The mechanical performance of PIE arises from the cooperative roles of flexible TTE soft segments, reversible hydrogen bonding, dynamic imine exchange, and the highly branched dynamic network, which respectively provide chain mobility, energy dissipation, stress relaxation, and efficient stress transfer, thereby enabling high stretchability, wide mechanical tunability, and durability under large deformation.

Additional tests were conducted to further evaluate environmental and mechanical stability. PIE2 exhibited low swelling ratios of 5.64% in water and 6.79% in artificial sweat after 72 h and maintained film integrity after 14 d of immersion (Fig. S8), indicating good stability under mild aqueous and sweat-like conditions. After exposure to 80% relative humidity, PIE2 still retained a tensile strength of 1.23 MPa and an elongation at break of 544% (Fig. S9), demonstrating that the elastomer maintained sufficient stretchability and mechanical integrity under humid conditions. Moreover, PIE2 withstood repeated stretching to 400% strain for 10 cycles without fracture (Fig. S10), confirming its mechanical durability under large cyclic deformation.

### Supramolecular structure and mechanism of PIE

**Fig. 4.**
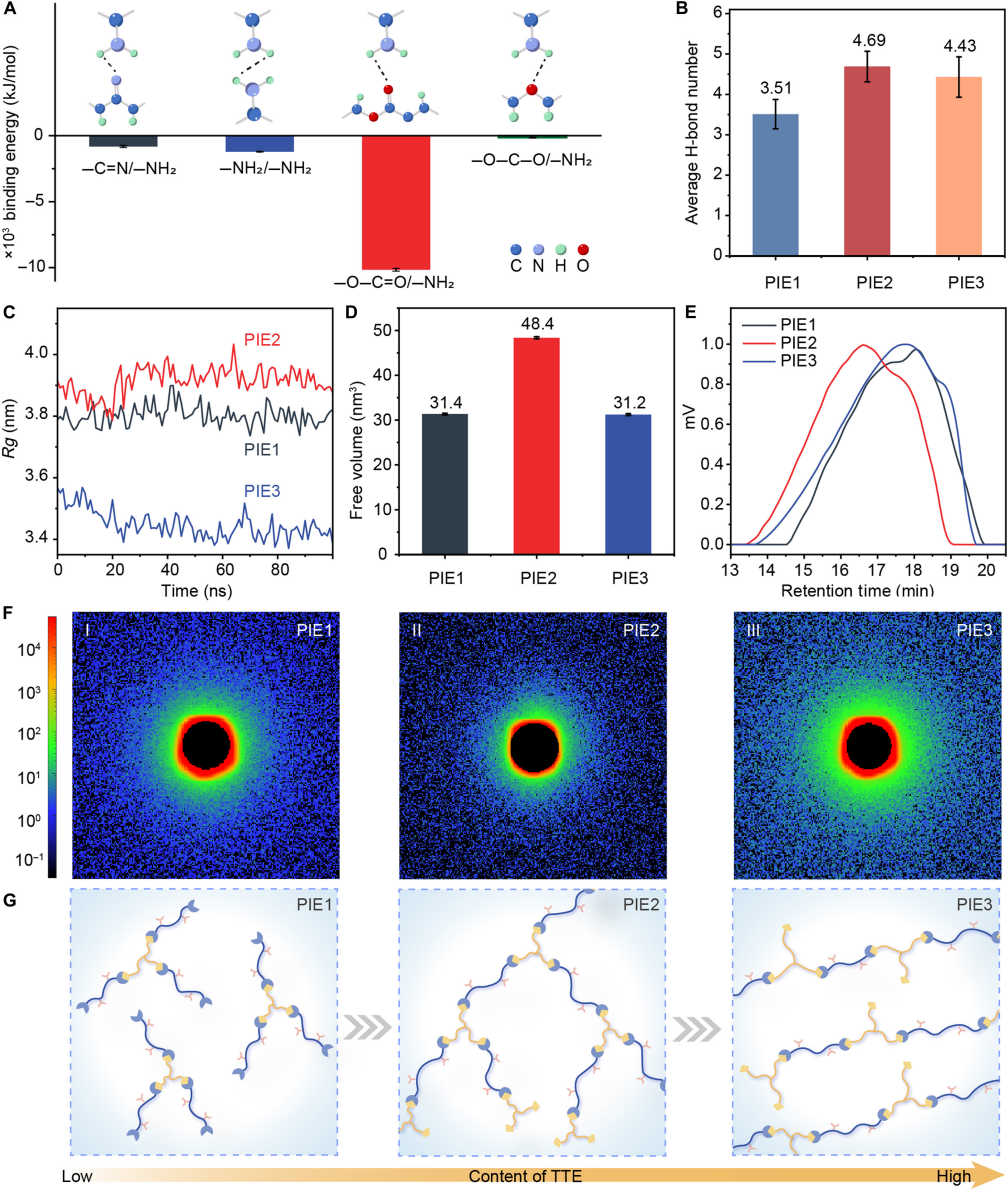
Supramolecular structure and mechanism of polyimine elastomer (PIE). (A) Binding energy of different hydrogen bonds calculated by molecular dynamics (MD). (B) Number of hydrogen bonds from MD simulations. (C) Radius of gyration (*Rg*) of PIE. (D) Free volume (FV) of PIE. (E) Gel permeation chromatography (GPC) elution curves of PIE. (F) Two-dimensional small-angle x-ray scattering (2D SAXS) patterns of PIE. (G) Schematic illustration of the supramolecular architecture of PIE.

The use of DTP, with its 2 aldehyde termini and extended chain structure, facilitates more effective connections with TTE and promotes the formation of a highly branched network, thereby enabling a broad and tunable mechanical window. To investigate the intrinsic structural features and interaction mechanisms within the material, molecular dynamics (MD) simulations were performed on the PIE systems. In the MD simulations, 3 PIE systems, corresponding to PIE1, PIE2, and PIE3, were constructed (Fig. S11), each containing 8 randomly arranged polymer chains within the simulation cell to replicate the equilibrium conformation of the actual polymers. The hydrogen-bonding interactions, which play a crucial role in determining the mechanical properties of the polymer, mainly include 4 types in PIE: amino–ester (O–C=O···–NH_2_), amino–imine (–C=N···–NH_2_), amino–amino (–NH_2_···–NH_2_), and amino–ether (O–C–O···–NH_2_) (Fig. 4A). The binding energy calculations show that the amino–ester interaction (O–C=O···–NH_2_) possesses the highest binding strength, highlighting the critical role of ester groups in the highly branched PIE network. In Fig. 4B, PIE2 displays the highest number of hydrogen bonds and the most robust internal interactions among the series. It initially rises with the increasing number of TTE donors but declines at excess levels (PIE3) as the limited acceptor sites of DTP and inhibited chain branching become dominant.

In the MD simulations, the radius of gyration (*Rg*) and free volume (FV) were calculated to elucidate the influence of dynamic interactions and highly branched networks. The *Rg* values (Fig. 4C) of PIE1, PIE2, and PIE3 were 3.773, 3.895, and 3.443 nm, respectively, exhibiting an initial increase followed by a decrease with increasing TTE content. Corroborating this trend, the FV values for the series were 31.4, 48.4, and 31.2 nm^3^ for PIE1, PIE2, and PIE3, respectively (Fig. 4D). The simultaneous expansion of *Rg* and FV in PIE2 suggests enhanced chain branching and spatial extension, which favor the formation of a more interconnected highly branched network and ultimately contribute to its superior stretchability. In contrast, PIE3 exhibits the smallest *Rg* and FV among the 3, reflecting reduced branching density and weaker hydrogen-bonding interactions, which together result in partial linearization and a pronounced loss of mechanical strength. Excessive TTE content thus suppresses branching and molecular expansion, producing a more linear architecture. Randomly captured molecular snapshots (Fig. S12) provide direct structural evidence of this trend, showing a more expanded and loosely packed network in PIE2.

Gel fraction and swelling measurements (Table S3) were performed to experimentally evaluate the network integrity of PIE1 to PIE3. PIE2 exhibited the highest gel fraction of 86.23% ± 1.18% and the lowest swelling ratio of 17.13% ± 2.28%, while PIE3 showed the opposite trend, indicating that PIE2 has the most effective network connectivity, whereas excessive TTE leads to a looser and more dynamically relaxable network. Rheological frequency-sweep measurements (Fig. S13) showed that PIE2 exhibited the highest storage modulus in the low- to medium-frequency region, together with elastic-dominated behavior characterized by *G*′ > *G*″ and tan *δ* < 1. In contrast, PIE3 showed tan *δ* > 1 in the medium- to high-frequency region, indicating dominant viscous dissipation and more pronounced chain relaxation due to excessive TTE-induced linear-like segments and reduced effective network connectivity. These rheological results indicate that PIE2 has the strongest effective elastic network. Consistently, the apparent effective cross-linking density estimated from dynamic mechanical analysis (DMA) follows the order PIE2 > PIE1 > PIE3 (Fig. S14 and Table S4), further confirming that PIE2 has highly branched network constraints. Gel permeation chromatography analysis further supports this trend (Fig. 4E). PIE2 shows the earliest elution peak and the highest polystyrene-calibrated apparent molecular weight among the 3 systems, with detailed values summarized in Table S5, indicating that PIE2 possesses the most developed branched structure.

Additionally, 2D small-angle x-ray scattering (SAXS) patterns reveal structural homogeneity, with the ring size distribution following the trend PIE3 > PIE1 > PIE2, further supporting PIE2’s more interconnected highly branched network architecture (Fig. 4F), consistent with the computed FV. A schematic model (Fig. 4G) illustrates that the expanded FV and cooperative dynamic interactions, including both hydrogen bonding and dynamic imine exchange, enable PIE2 to achieve superior stretchability and toughness. By contrast, PIE3 favors chain-extension-like growth over effective branching, leading to partial linearization, reduced branching density, diminished dynamic interactions, and increased chain slippage, ultimately resulting in a marked reduction in mechanical strength. Overall, the synergistic interplay of a highly branched architecture, abundant hydrogen-bonding interactions, and dynamic imine exchange underpins the exceptional and tunable mechanical performance of the elastomer.

### Thermal reprocessing and chemical recyclability of PIE

**Fig. 5.**
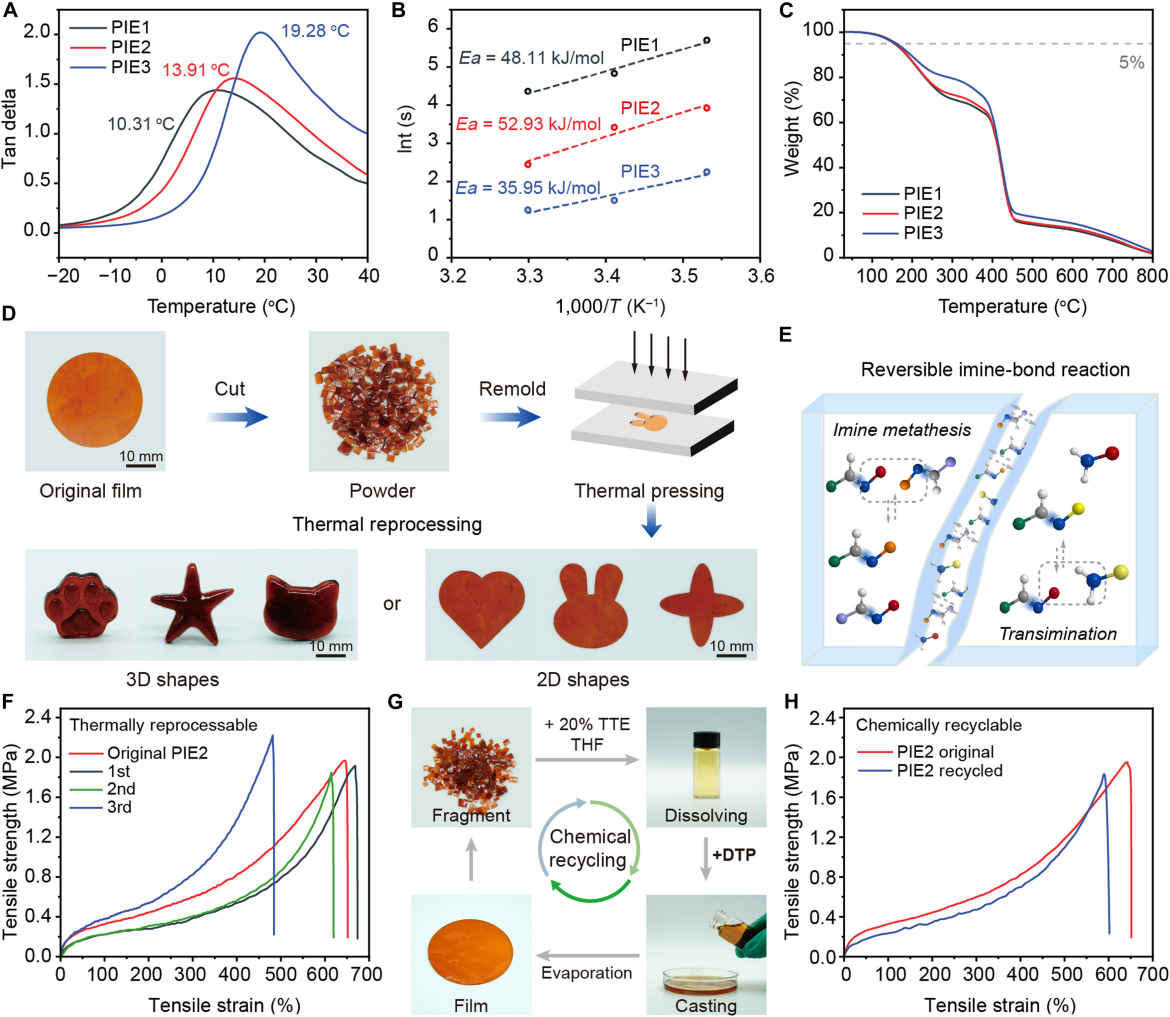
Thermal properties and recyclability of polyimine elastomer (PIE). (A) Tan *δ* curves obtained from dynamic mechanical analysis (DMA). (B) Relaxation times and activation energies (*Ea*) fitted using the Arrhenius equation. (C) Thermogravimetric curves of PIE. (D) Photographs of thermally reprocessed PIE2 formed into 2-dimensional (2D) and 3-dimensional (3D) structures. (E) Illustration of dynamic imine-bond exchange. (F) Tensile stress–strain curves of the original and hot-reprocessed PIE2. (G) Schematic diagram of the chemical recycling of PIE2. (H) Tensile stress–strain curves of the original and chemically recycled PIE2.

The highly branched architecture and dynamic bonds jointly endow the material with excellent thermal reprocessability and self-healing capability. The thermal and dynamic properties of PIE were investigated to elucidate their molecular mobility and reprocessability. DMA revealed that the glass-transition temperature (*Tg*) gradually increased from 10.31 °C (PIE1) to 19.28 °C (PIE3), indicating restricted chain mobility at higher TTE contents (Fig. 5A and Fig. S14). Polymers with *Tg* below room temperature exhibit soft and flexible networks, whereas those with *Tg* above room temperature display greater rigidity [48]. Network relaxation behavior (Fig. S15) exhibited an opposite trend: the relaxation time (1/*e*) decreased progressively from PIE1 to PIE3, reflecting the higher concentration of free amine groups that accelerate imine-bond exchange. Activation-energy analysis (Fig. 5B) further revealed values of 48.11 kJ mol^−1^ (PIE1), 52.93 kJ mol^−1^ (PIE2), and 35.95 kJ mol^−1^ (PIE3), with PIE2 showing the highest value, in agreement with its greater cross-linking density and molecular mass. These results confirm that the dynamic imine bonds enable rapid stress relaxation while maintaining structural stability. Thermal decomposition behavior revealed by thermogravimetric analysis also reflects the dynamic covalent nature of the network (Fig. 5C). As further evidenced by the derivative thermogravimetric profiles (Fig. S16), 2 distinct weight-loss regions were observed at 176 to 280 and 414 to 464 °C, corresponding to imine-bond cleavage followed by backbone degradation.

Benefiting from the reversibility of imine bonds, the materials displayed excellent thermal reprocessability: shredded PIE films could be completely reconstituted by hot-pressing at 100 °C for 15 min into 2D or 3D structures (Fig. 5D). Two reversible imine-bond exchange pathways are involved: imine-to-imine metathesis and transamination with free amines (Fig. 5E and Fig. S17). The recycled materials retained excellent mechanical performance (Fig. 5F). After the first and second hot-pressing cycles, the elongation remained nearly unchanged. A slight loss in ductility was observed after the third cycle, accompanied by increased tensile strength, which can be attributed to side reactions during repeated heating that generate highly branched polyamide structures (Fig. S18). Taking advantage of the reversible imine bonds, the PIE2 elastomer showed self-healing ability (Fig. S19). A dumbbell-shaped sample was cut and rejoined, followed by heating at 60 °C. The fracture interface was nearly completely healed, and the sample could be stretched without breaking. The mechanical properties progressively recovered during the healing process, reaching approximately 80% of their original strength through the cooperative action of dynamic imine exchange and hydrogen-bond reformation.

Beyond thermal reprocessability and self-healing capability, the dynamic imine and ester linkages also endow the material with chemical degradability and recyclability. PIE can be selectively degraded under basic or acidic conditions via ester-bond hydrolysis in tetrahydrofuran (THF)/NaOH (10%) and imine-bond dissociation in THF/HCl (10%) and completely dissolve within 15 min at room temperature (Fig. S20). Furthermore, the introduction of TTE induces the dissociation of imine bonds. The resulting depolymerized system can be recast after replenishing an equivalent amount of DTP, thereby enabling the chemical recycling of PIE (Fig. 5G). Analysis of the recycled samples (Fig. S21) further confirms, through their FT-IR spectra, that the original functional groups remain well-preserved after recovery. Utilizing these reversible reactions, the network can be chemically recycled, achieving more than 93% performance retention (Fig. 5H). The reversible imine network governs both the dynamic thermal responses and the tunable mechanical properties of PIE, highlighting the intrinsic connection between MD and macroscopic performance.

### Application of PIE as a stretchable conductor

**Fig. 6.**
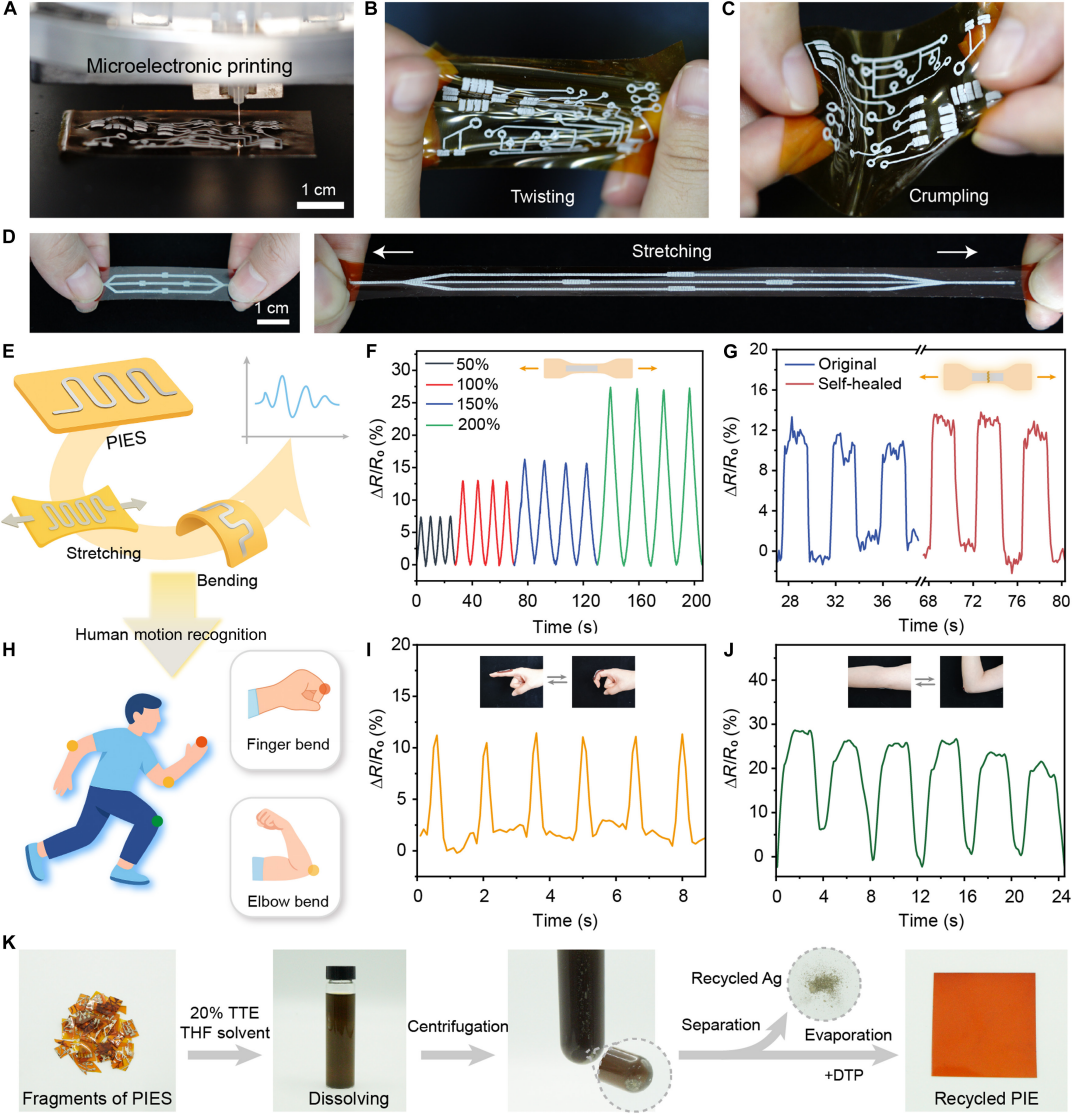
Fabrication and sensing performance of the polyimine elastomer sensor (PIES). (A to C) Images of polyimine elastomer (PIE) substrates patterned by microelectronic printing. (D) Photographs of screen-printed PIE substrates in the original and stretched states. (E) Schematic illustration of the sensing mechanism of the PIES. (F) Relative resistance changes under 50% to 200% strain. (G) Sensing performance before and after self-healing. (H) Schematic of human motion recognition applications. (I and J) Δ*R*/*R*_0_ signals during (I) finger and (J) elbow motion monitoring. (K) Flowchart of the PIES recycling process.

PIE possesses intrinsic softness and highly stretchable mechanical properties, along with excellent interfacial adaptability, making it a highly promising platform material for next-generation flexible and wearable electronics. As shown in Fig. 6A to C, conductive materials (e.g., silver paste and Ga–In alloy ink) can be precisely deposited onto the PIE surface through microelectronic printing, forming intricate and complex circuit patterns. Alternatively, screen printing enables the rapid fabrication of circuits (Fig. 6D). This enables the fabrication of printable PIE sensors (PIESs). The resulting PIES retains excellent flexibility and stretchability, indicating that the incorporation of conductive fillers does not compromise the intrinsic elasticity of the PIE network. Moreover, the PIE substrate exhibits intrinsic adhesive properties, allowing it to conformably attach to various surfaces such as metal, glass, paper, plastic, and human skin (Fig. S22). This adhesion capability is attributed to the presence of free amino groups and hydrogen-bond interactions within the polymer network.

Such versatility enables the seamless integration of the PIES into diverse stretchable and deformable electronic systems. Mechanical deformation of the PIES, including stretching or bending, induces corresponding changes in resistance, generating real-time response signals (Fig. 6E). The relative resistance change (Δ*R*/*R*_0_–*T*) curves under 50% to 200% strain (Fig. 6F) demonstrate stable and repeatable signal responses, even after multiple stretching cycles. To further quantify the sensing performance of the PIES, the gauge factor was calculated from the relationship between Δ*R*/*R*_0_ and applied strain. As shown in Fig. S23, Δ*R*/*R*_0_ increased nearly linearly with strain in the range of 50% to 200%, giving an average gauge factor of approximately 0.13, indicating a reliable strain-dependent resistance response of the sensor. In addition, the response and recovery behaviors were further evaluated (Fig. S24). The PIES exhibited rapid response and recovery times of 580 and 820 ms, respectively, demonstrating its capability for real-time strain monitoring.

The long-term sensing stability of the PIES was also investigated. As shown in Fig. S25, the sensor maintained a stable electrical response over 10,000 s, with no obvious signal attenuation or drift, confirming its excellent operational durability. This stable sensing behavior can be attributed to the intrinsic elasticity of the PIE matrix and the reversible dynamic imine bonds, which enable efficient stress relaxation and structural recovery during repeated deformation. Moreover, the PIES exhibited good humidity stability (Fig. S26), suggesting that the sensing signal was not markedly affected by environmental humidity and confirming its potential applicability under practical conditions. Remarkably, after being cut and self-healed, the sensor fully recovered its conductive pathway and maintained consistent electrical signals (Fig. 6G). To further evaluate the repeatability of the self-healing behavior, the sensing performance of the PIES after 3 consecutive cutting/healing cycles was tested. As shown in Fig. S27, the healed PIES still displayed stable and reproducible resistance responses, indicating that the dynamic imine network could repeatedly reconstruct the polymer matrix and facilitate the recovery of conductive pathways. These results confirm the robust self-healing capability and sensing reliability of PIES. To assess the feasibility of practical applications, the PIES was attached to human joints, such as the finger and elbow (Fig. 6H to J). During repeated bending, the Δ*R*/*R*_0_–*T* curves showed nearly identical waveforms and amplitudes, demonstrating excellent sensitivity and durability for detecting human motion. After use, the PIES fragments are dissolved in a THF solution containing 20% TTE, enabling centrifugation-assisted recovery of conductive Ag, followed by solvent evaporation to regenerate a PIE film (Fig. 6K). The regenerated PIE film was further fabricated into a recycled PIES, and its sensing performance was evaluated. As shown in Fig. S28, the recycled PIES retained a stable and repeatable resistance response during cyclic deformation, indicating that the dynamic imine chemistry endowed the PIES with excellent recyclability while preserving its sensing functionality. This closed-loop process highlights the recyclability and material circularity of the PIES enabled by dynamic imine chemistry. This confirms the PIES’s great potential in wearable health monitoring and motion recognition applications. Owing to its intrinsic stretchability, self-healing capability, and interfacial adaptability, the PIES hold great promise for applications in flexible electronics, stretchable sensors, and wearable electronic devices.

## Conclusion

In summary, we have developed a highly stretchable, self-healing, and recyclable PIE from renewable lignocellulose via a highly dynamic covalent branched network. Simply by adjusting the DTP/triamine feeding ratio, we directly governed the PIE’s branching structure, thereby modulating its internal dynamic imine/hydrogen bonding and FV to yield a low Young’s modulus (0.42 to 2.27 MPa) and tunable stretchability (314.1% to 1,113.3%). This tailored branched architecture, synergizing with the dynamic imine bonds, provides the material with excellent thermal reprocessability, self-healing capability, and chemical recyclability. Additionally, the PIE substrate can be seamlessly integrated with conductive materials through microelectronic or screen-printing techniques, forming printable PIE-based sensors that exhibit reliable electrical responsiveness, interfacial adhesion, and recyclability. This work establishes a sustainable approach for creating multifunctional elastomers from biomass, offering valuable design insights for future bio-based materials in flexible electronics and smart healthcare applications.

## Materials and Methods

### Materials

VA, AA, TTE (average *M*_n_ ≈ 440), *N*,*N*-dimethyl-4-pyridinamine (DMAP), *N*-(3-dimethylaminopropyl)-*N*′-ethylcarbodiimide hydrochloride (EDC), hydrochloric acid (HCl, 30%), sodium hydroxide (NaOH), deuterated dimethyl sulfoxide (DMSO-*d*_6_), hexafluoroisopropanol (HFIP), *N*,*N*-dimethylacetamide (DMAc), THF, and dimethylformamide (DMF) were all purchased from Aladdin Co., Ltd. (China). Conductive ink (Elasink 990) was obtained from Zhirou Technology Co., Ltd. (Beijing, China). Conductive silver paste (KZ-P55) was obtained from Kezhizhu Electronic Technology Co., Ltd. (Chongqing, China). All chemicals were used as received without further purification.

### Preparation of DTP

VA (2.00 g, 13.14 mmol) and AA (0.96 g, 6.57 mmol; 2:1 molar ratio) were dissolved in DMF, followed by the addition of DMAP (3.21 g) and EDC (2.51 g), each used in an equimolar amount relative to VA. The reaction mixture was stirred and allowed to react at room temperature for 4 h. After completion, the reaction was quenched by adding excess distilled water to precipitate the product. The solid was collected by vacuum filtration and dried under vacuum at room temperature to obtain the DTP powder. DTP was obtained as a white powder in 66.7% yield, exhibiting a *T*_m_ of 133 °C, a *T*_5%_ of 227 °C, and a *T*_max_ of 351 °C.

### Preparation of PIEs

DTP (1.0 g) was dissolved in DMAc under stirring. TTE (0.7 g) was then added, and the mixture was allowed to react at 80 °C for 4 h to form the precursor solution. PIE samples with different feed ratios were prepared by varying the DTP:TTE molar ratio (PIE1 = 3:2, PIE2 = 6:5, and PIE3 = 1:1). The resulting solution was poured into a mold and dried under vacuum. After drying, the freestanding PIE films were peeled from the mold.

### Preparation of the printable PIES

The PIES was fabricated using a microelectronic printer (Scientific 3A, Prtronic, Shanghai, China) operated in dispensing mode. A 1-mm-thick PIE substrate was used during printing. The printer was equipped with a needle of 0.21-mm inner diameter and operated at 150 psi at a printing speed of 1 mm s^−1^. After printing, the sample was dried at room temperature for 30 min. The conductive pathways were activated by stretching the dried substrate to yield the stretchable PIES. Alternatively, the ink could be deposited onto the PIE substrate by screen printing.

### Chemical and structural characterization

The chemical structure of DTP was confirmed by ^1^H NMR (Bruker Avance III HD 400 MHz, Germany) using DMSO-*d*_6_ as the solvent. Functional groups of DTP, PIE, and recycled PIE were analyzed by FT-IR spectroscopy (Nicolet iN10, Thermo Fisher Scientific, USA) over 4,000 to 400 cm^−1^ with 32 scans. Variable-temperature FT-IR spectra were recorded on an INVENIO S spectrometer (Bruker, Germany) from 30 to 100 °C at 5 °C intervals. X-ray photoelectron spectroscopy (K-Alpha, Thermo Fisher Scientific, UK) and x-ray diffraction (SmartLab 9 kW, Rigaku, Japan) were used to characterize the surface composition and crystalline structure, respectively. The apparent molecular weight of PIE was determined by gel permeation chromatography (PL-GPC 50, Agilent, USA) using HFIP as the eluent (0.1 mg ml^−1^, 25 °C, 1.0 ml min^−1^), with calibration against polystyrene standards. Rheological properties were measured by a rotational rheometer (HAAKE MARS 60, Thermo Fisher Scientific, Germany). Morphology was observed by scanning electron microscopy (JSM-7500F, JEOL, Japan). Two-dimensional SAXS patterns were collected using a Xeuss 3.0 system (Xenocs, France) equipped with a Cu x-ray source (*λ* = 1.54 Å, 8 keV).

### Thermal and mechanical characterization

The thermal stability of PIE was evaluated by thermogravimetric analysis (Discovery TGA 550, TA Instruments, USA) from 30 to 800 °C at a heating rate of 20 °C min^−1^ under nitrogen. DMA (Q880, TA Instruments, USA) was performed to determine the storage modulus (*E*′), loss modulus (*E*″), and loss tangent (tan *δ*) from −50 to 40 °C at 1 Hz with a strain amplitude of 0.1%. Stress–relaxation measurements were conducted by equilibrating samples at the target temperature for 3 min, applying 1% strain, and monitoring stress decay for 1 h. The mechanical properties of PIE, TPA-PIE, and recycled PIE (50 × 5 × 1 mm^3^) were measured using a universal testing machine (Instron 5569, USA) at a tensile rate of 15 mm min^−1^. Cyclic tensile tests were carried out at 300% strain and 100 mm min^−1^ for 5 cycles, followed by 24-h recovery before retesting. Puncture resistance was evaluated using a SANS UTM5300H instrument with a needle probe at 15 mm min^−1^.

### Self-healing characterization

Self-healing tests were performed on dumbbell-shaped specimens. The specimens were cut at the center, rejoined by contacting the freshly exposed surfaces, and healed at 60 °C for 24 h without external pressure. The healed specimens were subsequently characterized by tensile testing. The healing efficiency (*η*) was calculated from the recovery of elongation at break according to

$$\eta \left(\%\right)=\frac{\varepsilon_{h}}{\varepsilon_{0}}\times 100\%$$ (1)

where *ε*_0_ and *ε_h_* represent the elongations at break of the original sample and the sample healed for 24 h, respectively.

### Electrical sensing tests

A PIES conductor (40 × 10 × 1 mm^3^) was used for fabricating flexible motion sensors. The 2 ends of the conductor were connected to copper wires and subjected to stretching, bending, and joint-mounted deformation. Relative resistance changes were recorded using an electrochemical workstation (CHI 660D, Shanghai Chenhua Instrument Co., Ltd., China). The relative resistance variation (Δ*R*/*R*_0_) was calculated according to

$$\Delta R/R_{0}=\frac{R-R_{0}}{R_{0}}\times 100\%$$ (2)

where *R* and *R*_0_ represent the resistances of the stretched and undeformed states, respectively.

### MD simulations

All-atom MD simulations of networks with different branching degrees were conducted using GROMACS 2024.1 with the general Amber force field (GAFF2). Each system contained 8 randomly distributed molecules, followed by energy minimization and 100-ns equilibration under the NPT ensemble (298 K, 1 bar, 2 fs). The V-rescale and Berendsen algorithms were employed to control temperature and pressure, respectively, and VMD 1.9.3 was used for visualization. After equilibrium was achieved, statistical analyses were performed on hydrogen bonding, cohesive energy, radius of gyration (*Rg*), and FV. The binding energy of each hydrogen bond was represented by the electrostatic interaction between specific functional groups.

## Data Availability

The data that support the findings of this study are available from the corresponding authors upon reasonable request.
