# Supplementary material for: Stretchable, Self-Healing, and Processable Bioelastomers Enabled by Highly Branched Cross-Linking Networks for Flexible Electronics
Source: Research (Wash D C). 2026 Aug 5;9:1391. doi: 10.34133/research.1391 (PMC13438028; doi:10.34133/research.1391)
Supplement: Supplementary file 1 [file research.1391.f1.docx]

**Supporting Materials**

**Stretchable, self-healing, and processable bioelastomers enabled by highly branched cross-linking networks for flexible electronics**

Yuan Liu^1^, Xin Xu^1^, Jinsong Sun^1^, Aojing Xue^1^, Jianing Liu^1^, Yuhan Lou^1^, Jiajun Liu^1^, Shi Liu^1^, Dan Sui^2^, Zhiyi Hou^2^, Gegu Chen^3^, Luyao Wang^1^, Yongzhuang Liu^1^, Dawei Zhao^1,4*^, Haipeng Yu^1*^, Qinqin Xia^1*^

1.	State Key Laboratory of Woody Oil Resources Utilization, Northeast Forestry University, Harbin 150040, China

2.	Analysis and Testing Center, Northeast Forestry University, Hexing Road 26, Harbin 150040, P.R. China

3.	MOE Engineering Research Center of Forestry Biomass Materials and Bioenergy, Beijing Forestry University Beijing, 100083, China

4.	Key Laboratory on Resources Chemicals and Materials of Ministry of Education, Shenyang University of Chemical Technology, Shenyang 110142, P. R. China

*Corresponding:

[2018xiaqinqin@nefu.edu.cn](mailto:2018xiaqinqin@nefu.edu.cn)

[yuhaipeng20000@nefu.edu.cn](mailto:yuhaipeng20000@nefu.edu.cn)

[daweizhao@syuct.edu.cn](mailto:daweizhao@syuct.edu.cn)

**Figures**

**Fig. S1.** Synthesis of DTP. (a) Digital image of DTP powder. (b) ¹H NMR spectrum of DTP in DMSO-d₆.

**Fig. S2.** XPS spectra of PIE2. (a) Survey spectrum. (b) C 1s spectrum. (c) N 1s spectrum.

**Fig. S3.** SEM images of PIE2.

**Fig. S4.** (a) XRD pattern of PIE2. (b) 2D WAXS pattern of PIE2.

**Fig. S5.** FT-IR spectra of PIE1–PIE3.

**Fig. S6.** Force–displacement curves of PIE1–PIE3.

**Fig. S7.** Cyclic tensile curves of PIE2 under cyclic loading.

**Fig. S8.** Stability of PIE in water and artificial sweat. (a) Swelling ratios of PIE2 after immersion in water and artificial sweat for 72 h. (b) Photographs of PIE2 samples immersed in water and artificial sweat before and after 14 days.

**Fig. S9.** Tensile stress–strain curves of PIE2 before and after exposure to 80% relative humidity.

**Fig. S10.** Cyclic tensile stress–strain curves of PIE2 under repeated stretching to 400% strain for 10 cycles.

**Fig. S11.** Snapshots of the all-atom MD simulations of the structures of PIE1–PIE3.

**Fig. S12.** All-atom MD snapshots showing the network structures of PIE1–PIE3.

**Fig. S13.** Rheological characterization of PIE1–PIE3. (a) Frequency-dependent storage modulus (G′) and loss modulus (G″). (b) tan δ values as a function of angular frequency.

**Fig. S14.** Storage modulus curves of PIE1–PIE3 as a function of temperature measured by DMA.

**Fig. S15.** Stress-relaxation behavior of PIE. (a–c) Stress-relaxation curves of PIE at 10 °C, 20 °C, and 30 °C. (d–f) Relaxation times and activation energies (Ea) fitted to the Arrhenius equation.

**Fig. S16.** DTG curves of PIE1–PIE3.

**Fig. S17.** Mechanisms of exchangeable imine bonds.

**Fig. S18.** FT-IR spectra of PIE2 and hot-reprocessed PIE2.

**Fig. S19.** Self-healing property of PIE. (a) Photograph of the PIE2 sheet that was cut in half and healed for 24 h. (b) Stress–strain curves of the PIE2 sheet healed at 60 °C for different durations.

**Fig. S20.** Chemical degradation of PIE under different conditions. (a) PIE degraded in 20 wt.% TTE in THF. (b) PIE degraded in 1 M HCl in THF. (c) PIE degraded in 1 M NaOH in THF, demonstrating the reversibility of imine bonds.

**Fig. S21.** FT-IR spectra of PIE2 and chemically recycled PIE2.

**Fig. S22.** Photographs of PIE2 showing excellent adhesion to various representative substrates.

**Fig. S23.** (a) Cyclic strain-sensing performance of the sensor at strains of 50%, 100%, 150%, and 200%. (b) Relative resistance change (ΔR/R₀) versus strain used for gauge factor (GF) calculation.

**Fig. S24.** Response and recovery times of the PIES.

**Fig. S25.** Stability of the sensor during 10,000 s of stretching-releasing cycles.

**Fig. S26.** Relative resistance change of the PIES sensor under 150% strain at 80% relative humidity.

**Figure S27.** Electrical stability of PIES after repeated healing processes. (a) Relative resistance response of the original and self-healed PIES under cyclic deformation. (b) Relative resistance response of PIES after three consecutive healing cycles.

**Figure S28.** Sensing performance of the recycled PIES under 200% tensile strain.

**Tables**

**Table S1.** Signs of cross-peaks from synchronous and asynchronous 2D-COS spectra of the PIE during heating.

|  | 1625 | 1673 | 1748 | 1761 | 3450 |
| --- | --- | --- | --- | --- | --- |
| 1625 |  | **+** | **+** | **+** | + |
| 1673 |  |  | **+** | **+** | + |
| 1748 |  |  |  | **-** | + |
| 1761 |  |  |  |  | + |
| 3450 |  |  |  |  |  |

*(“+” indicates Φ × Ψ > 0; “−” indicates Φ × Ψ < 0.)*

Temperature-dependent 2D correlation infrared spectroscopy (2D-COS-IR) was conducted to further elucidate the thermally induced evolution of hydrogen-bonding interactions in PIE. In the synchronous map, negative cross-peaks at 1625 × 1673 cm⁻¹and 1625 × 1761 cm⁻¹ indicate that the attenuation of hydrogen-bonded C=N···H–N interactions occurs concurrently with the enhancement of free C=N and C=O absorptions, suggesting a competitive redistribution of hydrogen donors between imine and carbonyl acceptors. Positive correlations at 1761 × 1748 cm⁻¹ reveal cooperative variations between carbonyl vibrations, while the positive cross-peak at 1748×3450 cm⁻¹ implies that the formation of C=O···H–N hydrogen bonds accompanies the gradual release of N–H groups. Analysis of the asynchronous map, following Noda’s rule, establishes the sequential order of spectral changes as 1625 → 1673 → 1761 → 1748 → 3450 cm⁻¹ (→ means prior to or earlier than), i.e. ν(C=N) (hydrogen-bonded) → ν(C=N) (free) → ν(C=O) (free) → ν(C=O) (hydrogen-bonded) → ν(N–H), indicating that hydrogen-bonded C=N···H–N interactions dissociate first, followed by the strengthening of free C=N, the rearrangement of carbonyl absorptions, and the eventual formation of new C=O···H–N linkages, with free N–H vibrations stabilizing at elevated temperatures. Overall, these results confirm the coexistence of two types of hydrogen bonds C=N···H–N and C=O···H–N in the PIE system, with the latter exhibiting stronger binding strength and dominating the thermally driven reconfiguration of the hydrogen-bonding network.

**Table S2.** Comparison of the mechanical properties of PIE, petroleum-based elastomers, and bio-based elastomers.

| **Samples** | **Stress (MPa)** | **Strain (%)** | **Young’s modulus (MPa)** | **Ref.** |
| --- | --- | --- | --- | --- |
| PIE1 | 1.14 | 314.1 | 0.42 | This work |
| PIE2 | 1.95 | 640.6 | 2.27 |  |
| PIE3 | 0.61 | 1113.3 | 2.17 |  |
| PDMS-5000/AL | 0.24 | 124 | 0.35 | Ref [40] |
| PDMS-500/AL 90wt% | ≈0.076 | 236 | 0.031 |  |
| PDMS | 0.40 ± 0.01 | 210 ± 16 | 0.61 ± 0.01 | Ref [41] |
| CNT0.5/Ecoflex | ≈1.25 | ＞800 | 0.11 | Ref [10] |
| Ecoflex | ≈0.18 | ≈920 | - | Ref [42] |
| VegPU1 | 0.66 | 797 | - | Ref [12] |
| VegPU4 | 1.67 | 205 | - |  |
| A2L | ≈1.1 | ≈200 | - | Ref [13] |
| A1L | ≈0.7 | ≈300 | - |  |
| A0L | 0.14 | 114 | - |  |
| SC10 | ≈0.8 | ≈600 | - | Ref [5] |
| SC5 | ≈0.73 | ≈570 | - |  |
| SC1 | ≈0.4 | ≈462 | - |  |
| DCIE-5 | 0.48 | ≈927 | 0.3 | Ref [24] |
| DCIE-3 | ≈0.413 | ≈1000 |  |  |
| TVn-p | 2.9±0.78 | 87±18 |  | Ref [43] |
| P2 | ≈1.1 | 120±25 | 1.6±1.0 | Ref [44] |
| ED-0.75 | 0.8 | 65 | - | Ref [45] |
| ED-0.8 | 0.7 | 83 | - |  |
| ED-0.85 | 0.62 | 102 | - |  |
| Cell1.8-PVA60 | 0.2±0.02 | 955±78 | - | Ref [46] |
| Cell1.9-PVA90 | 1.6±0.2 | 732±45 | - |  |
| Cell1.2-PVA120 | 0.8±0.1 | 847±53 | - |  |

**Table S3.** Swelling ratio and gel fraction of PIE1–PIE3.

| **Type** | **Swell ratio (%)** | **Gel fraction (%)** |
| --- | --- | --- |
| PIE1 | 23.44±0.63 | 82.31±1.42 |
| PIE2 | 17.13±2.28 | 86.23±1.18 |
| PIE3 | 25.56±1.07 | 73.39±1.65 |

The swelling ratio and gel fraction of PIE samples were measured using acetone as the swelling/extraction solvent. Briefly, dried PIE samples were weighed to obtain the initial mass (W₀) and then immersed in acetone at room temperature (~25 °C) for 2 days. After swelling, the samples were gently removed, and the surface solvent was wiped off with filter paper before weighing to obtain the swollen mass (W_s_). The swelling ratio was calculated according to the following equation:

$$Swelling ratio(\%)=\frac{W_{s}-W_{0}}{W_{o}}\times100\%$$

Subsequently, the swollen samples were dried in a vacuum oven at 60 °C to a constant weight to obtain the mass of the dried insoluble residue (W_d_). The gel fraction was calculated according to the following equation:

$$Gel fraction\left( \% \right)=\frac{W_{d}}{W_{0}}\times100\%$$

where W₀, Wₛ, and W_d_ represent the initial dry mass, swollen mass, and dried insoluble residue mass of the PIE sample, respectively.

**Table S4.** Cross-linking degree test of PIE.

| **Samples** | **Ec (MPa)** | **Tg (^o^C)** | **Tc (K)** | **Ed (MPa)** | **ρ (mol/m^3^)** |
| --- | --- | --- | --- | --- | --- |
| PIE1 | 2347.9 | 10.31 | 313.46 | 0.62 | 79.34 |
| PIE2 | 3435.9 | 13.97 | 317.12 | 0.78 | 98.48 |
| PIE3 | 3036.1 | 19.28 | 322.43 | 0.39 | 48.87 |

Ec is the storage modulus at -50 ^o^C, Tg is the glass transition temperature, Ed is the storage modulus at (Tg + 30 ^o^C), and ρ is the crosslinking density.

The cross-linking density (ρ) was calculated by the following equation:

$$\rho=\frac{E_{d}}{3RT_{c}}$$

where Tc is the absolute temperature defined as Tg + 30 ^o^C, Ed is the storage modulus at a temperature of Tc, and R is the universal gas constant (8.314 J·mol^-1^·K^-1^).

**Table S5.** GPC results of the PIE1–PIE3

|  | **Mn** | **Mw** | **PDI** |
| --- | --- | --- | --- |
| PIE1 | 6725 | 12946 | 1.93 |
| PIE2 | 12625 | 45939 | 1.96 |
| PIE3 | 7153 | 15901 | 2.22 |
